# Supplementary material for: Output variability across animals and levels in a motor system
Source: eLife. 2018 Jan 18;7:e31123. doi: 10.7554/eLife.31123 (PMC5773184; doi:10.7554/eLife.31123)
Supplement: Figure 3—source data 1. — Data in grey boxes are plotted in Figure 3. [file elife-31123-fig3-data1.docx]

Figure 3–source data 1 Wenning, Norris, Günay, Kueh & Calabrese

**Cycle-to-Cycle Variances***

| *Data shown on Figure 3A* | **CPG Pattern**  **(N = 26)** | | **Motor Pattern**  **(N = 33)** | | **Beat pattern**  **(N = 12)** | |
| --- | --- | --- | --- | --- | --- | --- |
| Peristaltic  Coordination | **Left** | **Right** | **Left** | **Right** | **Left** | **Right** |
| Cycle 1 | 0.6 ± 0.2 | 0.7 ± 0.6 | 0.9 ± 0.6 | 0.9 ± 0.7 | 1.1 ± 0.9 | 1.2 ± 1.2 |
| Cycle 2 | 0.7 ± 0.5 | 0.5 ± 0.3 | 0.7 ± 0.5 | 0.7 ± 0.4 | 0.7 ± 0.3  (N = 9) | 0.8 ± 0.4  N = 8) |
| HN(R,4)-HN(L,4)  Cycle 1 | 0.3 ± 0.2 | |  |  |  |  |
| Synchronous  Coordination | **Left** | **Right** | **Left** | **Right** | **Left** | **Right** |
| Cycle 1 | 0.3 ± 0.3 | 0.4 ± 0.5 | 0.6 ± 0.8 | 0.4 ± 0.3 | 1.6 ± 1.6 | 1.6 ± 2.3 |
| Cycle 2 | 0.3 ± 0.2 | 0.4 ± 0.4 | 0.4 ± 0.4 | 0.4 ± 0.3 | 0.8 ± 0.8  (N= 9) | 1.2 ± 0.6  (N = 8) |

*****Angular variance *s^2^* in 10^-3^ phase squared
