# Supplementary material for: Output variability across animals and levels in a motor system
Source: eLife. 2018 Jan 18;7:e31123. doi: 10.7554/eLife.31123 (PMC5773184; doi:10.7554/eLife.31123)
Supplement: Figure 4—source data 1. — (TOP) Note that intersegmental Δϕ differs significantly between the CPG pattern and the motor pattern in both coordinations and on both sides. (BOTTOM) Note that population variances are lowest in the motor pattern in both coordinations and on both sides. Data in grey boxes are plotted in Figure 4. [file elife-31123-fig4-data1.docx]

Figure 4–source data 1 Wenning, Norris, Günay, Kueh & Calabrese

**Intersegmental Phase Differences Δ* ***

| *Data shown on*  *Figure 4B* | **CPG Pattern**  **(N = 26)** | | **Motor Pattern**  **(N = 33)** | | **Beat pattern**  **(N = 12)** | |
| --- | --- | --- | --- | --- | --- | --- |
| Peristaltic Coordination | **Left** | **Right** | **Left** | **Right** | **Left** | **Right** |
| Δ*φ* ± SD | 0.225 ± 0.06 | 0.216 ± 0.06 | 0.130 ± 0.05  p < 0.001 | 0.135 ± 0.05 | 0.213 ± 0.06 | 0.204 ± 0.07 |
| *Different within side and mode? *** | p < 0.001 | | | | | |
| Synchronous Coordination | **Left** | **Right** | **Left** | **Right** | **Left** | **Right** |
| Δ*φ* ± SD | -0.076 ± 0.06 | -0.085 ± 0.04 | -0.032 ± 0.04 | -0.035 ± 0.04  p = 0.001 | -0.034 ± 0.07 | -0.030 ± 0.1 |
| *Different within side and mode?* ****** | p < 0.001 | | | | | |

***** Δ* *in phase units

****** unpaired t-Test

**Population Variances***

| *Data shown on Figure 4C* | **CPG Pattern**  **(N = 26)** | | **Motor Pattern**  **(N = 33)** | | **Beat pattern**  **(N = 12)** | |
| --- | --- | --- | --- | --- | --- | --- |
| Peristaltic Coordination | **Left** | **Right** | **Left** | **Right** | **Left** | **Right** |
| Cycle 1 | 2.9 | 3.4 | 2.1 | 2.4 | 2.8 | 4.2 |
| Vector length | 0.942251 | 0.932753 | 0.959436 | 0.952304 | 0.943727 | 0.91779 |
| Confidence Intervals | 1.8 – 5.0 | 2.3 – 5.7 | 1.2 – 3.5 | 1.6 – 3.9 | 1.7 – 4.9 | 2.3 – 7.9 |
| Synchronous Coordination | **Left** | **Right** | **Left** | **Right** | **Left** | **Right** |
| Cycle 1 | 3.2 | 1.8 | 1.5 | 1.4 | 4.0 | 7.9 |
| Vector length | 0.937669 | 0.96368 | 0.969384 | 0.971506 | 0.921451 | 0.844794 |
| Confidence Intervals | 1.9 – 6.2 | 1.1 – 3.2 | 1.2 – 2.1 | 1.0 – 2.4 | 2.2 – 6.8 | 4 – 16.0 |

*****Angular variance *s^2^* and confidence intervals after bootstrapping in 10^-3^ phase squared
