# Supplementary material for: Output variability across animals and levels in a motor system
Source: eLife. 2018 Jan 18;7:e31123. doi: 10.7554/eLife.31123 (PMC5773184; doi:10.7554/eLife.31123)
Supplement: Figure 6—source data 1. — Note that all variances (both coordinations, both sides) are smaller than those of 10,000 scrambled populations. Data in grey boxes are plotted on Figure 6. [file elife-31123-fig6-data1.docx]

Figure 6–source data 1 Wenning, Norris, Günay, Kueh & Calabrese

**Repetition Variances***

| *Data shown on Figure 6* | **CPG Pattern**  **(N = 24) **** | | **Motor Pattern**  **(N = 32) **** | | **Beat pattern**  **(N = 8/9) **** | |
| --- | --- | --- | --- | --- | --- | --- |
| Peristaltic Coordination | **Left** | **Right** | **Left** | **Right** | **Left** | **Right** |
| Δ* *of Switch cycles 1 & 2 differed in n of N experiments | 10 of 24 | 13 of 24 | 17 of 32 | 18 of 32 | 5 of 9 | 5 of 8 |
| Variance* of ΔΔ*φ* = Δ*φ*_cycle1_ - Δ*φ*_cycle2_ | 1.5 | 0.8 | 0.8 | 0.3 | 0.5 | 1.0 |
| Scrambling Test  (bootstrapping) | p < 0.001 | p < 0.001 | p < 0.001 | p <0.001 | p = 0.004 | p = 0.003 |
| Average \|ΔΔ*φ* \| (in phase units) | 0.028 | 0.023 | 0.021 | 0.016 | 0.020 | 0.029 |
| Synchronous Coordination | **Left** | **Right** | **Left** | **Right** | **Left** | **Right** |
| Δ* *of Switch cycles 1 & 2 differed in n of N experiments | 12 of 24 | 15 of 24 | 22 of 32 | 23 of 32 | 3 of 9 | 5 of 8 |
| Variance* of ΔΔ*φ* = Δ*φ*_cycle1_ - Δ*φ*_cycle2_ | 0.7 | 0.8 | 0.5 | 0.6 | 1.3 | 4.1 |
| Scrambling Test  (bootstrapping) | p < 0.001 | p <0.001 | p < 0.001 | p < 0.001 | p = 0.011 | p = 0.006 |
| Average \|ΔΔ*φ* \| (in phase units) | 0.019 | 0.020 | 0.017 | 0.019 | 0.028 | 0.052 |

*****Angular variance *s^2^* and confidence intervals after bootstrapping in 10^-3^ phase squared

****** Not all preparations had two full switch cycles (CPG pattern: 24 of 26; Motor pattern: 32 of 33; Beat pattern: 8 of 12, right side, 9 of 12, left side).

significant
