# Supplementary material for: Output variability across animals and levels in a motor system
Source: eLife. 2018 Jan 18;7:e31123. doi: 10.7554/eLife.31123 (PMC5773184; doi:10.7554/eLife.31123)
Supplement: Figure 7—source data 1. — Note that variances in synchronous coordination are smaller than those of 10,000 scrambled populations while those in peristaltic coordination are similar to the scrambled populations in the CPG pattern and the beat pattern, but smaller in the motor pattern. Data in grey boxes are plotted on Figure 7. [file elife-31123-fig7-data1.docx]

Figure 7–source data 1 Wenning, Norris, Günay, Kueh & Calabrese

**Bilateral Variances***

| *Data shown on Figure 7* | **CPG Pattern**  **(N = 26)** | **Motor Pattern**  **(N = 33)** | **Beat pattern**  **(N = 11) **** |
| --- | --- | --- | --- |
| Peristaltic Coordination | | | |
| Right Δ* *and Left Δ* *differed in n of N experiments | 20 of 26 | 29 of 33 | 8 of 11 |
| Variance* of ΔΔ*φ*  = Δ* *_Right_ - Δ*φ*_Left_ | 5.1 | 2.1 | 4.5 |
| Scrambling Test  (bootstrapping) | p = 0.18 | p = 0.001 | p = 0.15 |
| Average \|ΔΔ*φ* \|  (in phase units) | 0.059 | 0.040 | 0.056 |
| Synchronous Coordination | | | |
| Right Δ* *and Left Δ* *differed in n of N experiments | 19 of 26 | 28 of 33 | 9 of 11 |
| Variance* of ΔΔ*φ*  = Δ* *_Right_ - Δ*φ*_Left_ | 1.7 | 1.2 | 4.1 |
| Scrambling Test (bootstrapping) | p < 0.001 | p < 0.001 | p = 0.011 |
| Average \|ΔΔ*φ* \|  (in phase units) | 0.031 | 0.028 | 0.058 |

*****Angular variance *s^2^* and confidence intervals after bootstrapping in 10^-3^ phase squared

****** Bilateral recordings in 11 of 12 preparations.

significant; not significant
