# Supplementary material for: Output variability across animals and levels in a motor system
Source: eLife. 2018 Jan 18;7:e31123. doi: 10.7554/eLife.31123 (PMC5773184; doi:10.7554/eLife.31123)
Supplement: Transparent reporting form [file elife-31123-transrepform.docx]

***eLife’s* transparent reporting form**

We encourage authors to provide detailed information *within their submission* to facilitate the interpretation and replication of experiments. Authors can upload supporting documentation to indicate the use of appropriate reporting guidelines for health-related research (see [EQUATOR Network](http://www.equator-network.org/%20)), life science research (see the [BioSharing Information Resource](https://biosharing.org/)), or the [ARRIVE guidelines](http://www.plosbiology.org/article/info:doi/10.1371/journal.pbio.1000412) for reporting work involving animal research. Where applicable, authors should refer to any relevant reporting standards documents in this form.

If you have any questions, please consult our Journal Policies and/or contact us: [editorial@elifesciences.org](mailto:editorial@elifesciences.org).

**Sample-size estimation**

- You should state whether an appropriate sample size was computed when the study was being designed
- You should state the statistical method of sample size computation and any required assumptions
- If no explicit power analysis was used, you should describe how you decided what sample (replicate) size (number) to use

Please outline where this information can be found within the submission (e.g., sections or figure legends), or explain why this information doesn’t apply to your submission:

Thirty years of experience working with long-term recordings from isolated chains of ganglia in leeches taught us that ≥ 8 individuals are sufficient to determine average intersegmental phase differences across animals.

Within one animal, ≥ 7 (normally >10), each with 30 to fifty spikes, are sufficient to determine the average synaptic strength from a given interneuron to a given motor neuron in a given preparation, and ≥ 7 (normally >10) bursts are sufficient to determine the average segmental phase difference for that animal.

**Replicates**

- You should report how often each experiment was performed
- You should include a definition of biological versus technical replication
- The data obtained should be provided and sufficient information should be provided to indicate the number of independent biological and/or technical replicates
- If you encountered any outliers, you should describe how these were handled
- Criteria for exclusion/inclusion of data should be clearly stated
- High-throughput sequence data should be uploaded before submission, with a private link for reviewers provided (these are available from both GEO and ArrayExpress)

Please outline where this information can be found within the submission (e.g., sections or figure legends), or explain why this information doesn’t apply to your submission:

Figure 2 (suppl) subdivides our entire Project Database of in vitro recordings from over 150 animals collected from 9 years of work. Figure 2 (suppl) also describes our Intact Animal Database where we imaged the hearts bilaterally. Each group analyzed is given a name and a Figure 2 (suppl) designation, which are used in the text, so that it is unambiguous which animals were used. Clearly shown are the new preparations previously unreported in any publication in which we made bilateral recordings for this study. N refers to the number of different animals (preparations) used in the analyses, n refers to the number of cycles of the rhythmic activity recorded (Fig. 2A4,B4,C4). See section Results/Background. Handling outliers is explained in Methods.

**Statistical reporting**

- Statistical analysis methods should be described and justified
- Raw data should be presented in figures whenever informative to do so (typically when N per group is less than 10)
- For each experiment, you should identify the statistical tests used, exact values of N, definitions of center, methods of multiple test correction, and dispersion and precision measures (e.g., mean, median, SD, SEM, confidence intervals; and, for the major substantive results, a measure of effect size (e.g., Pearson's r, Cohen's d)
- Report exact p-values wherever possible alongside the summary statistics and 95% confidence intervals. These should be reported for all key questions and not only when the p-value is less than 0.05.

Please outline where this information can be found within the submission (e.g., sections or figure legends), or explain why this information doesn’t apply to your submission:

Statistics: ‘Statistics and Metrics’ in Methods

The p-values are on the figures, in the tables, and in the text.

(For large datasets, or papers with a very large number of statistical tests, you may upload a single table file with tests, Ns, etc., with reference to sections in the manuscript.)

**Group allocation**

- Indicate how samples were allocated into experimental groups (in the case of clinical studies, please specify allocation to treatment method); if randomization was used, please also state if restricted randomization was applied
- Indicate if masking was used during group allocation, data collection and/or data analysis

Please outline where this information can be found within the submission (e.g., sections or figure legends), or explain why this information doesn’t apply to your submission:

Figure 2 (supplement) describes the data base.

**Additional data files (“source data”)**

- We encourage you to upload relevant additional data files, such as numerical data that are represented as a graph in a figure, or as a summary table
- Where provided, these should be in the most useful format, and they can be uploaded as “Source data” files linked to a main figure or table
- Include model definition files including the full list of parameters used
- Include code used for data analysis (e.g., R, MatLab)
- Avoid stating that data files are “available upon request”

Please indicate the figures or tables for which source data files have been provided:

The raw data and the codes to analyze them are on datadryad.org and github.com. Accompanying the manuscript is a supplementary file containing the data which are the basis for all figures and tables (*Experimental Data and Analysis for All Figures.xlsx*).
